# A Single Center Initial Experience with Robotic-Assisted Minimally Invasive Coronary Artery Bypass Surgery (RA-MIDCAB)

**DOI:** 10.3390/jpm12111895

**Published:** 2022-11-12

**Authors:** Antonio Piperata, Olivier Busuttil, Jean-Luc Jansens, Thomas Modine, Mathieu Pernot, Louis Labrousse

**Affiliations:** 1 Department of Cardiology and Cardio-Vascular Surgery, Hopital Cardiologique de Haut-Leveque, Bordeaux University Hospital, 33604 Pessac, France; 2 Department of Cardiac Surgery, Erasme Hospital of Brussels, Belgium free University of Brussels (ULB), Hôpital Erasme, 1070 Brussels, Belgium

**Keywords:** robotic coronary artery bypass grafting, coronary artery revascularization, minimally invasive surgery

## Abstract

Background: Minimally invasive procedures have demonstrated their effectiveness in reducing the recovery times while ensuring optimal results and minimizing complications. Regarding the coronary artery surgical revascularization field, the evolution of techniques and technology is permitting new surgical strategies that are increasingly precise and suitable for each patient. We present an initial single center experience with a case series of patients successfully treated with combined robotic harvesting of the left internal mammary artery (LIMA) and minimally invasive direct coronary artery bypass graft (MIDCAB) for the anastomosis. Methods: We retrospectively reviewed the records of patients who underwent minimally invasive coronary artery revascularization with the use of two combined techniques at our Institution between January 2021 and October 2022. Results: A total of 17 patients underwent coronary artery bypass grafting with the described approach. The median cardiopulmonary bypass (CPB) and cross-clamp times were 83 min (76–115) and 38 min (32–58), respectively. The median intensive care unit (ICU) and hospital stay were 2 days (1–4) and 8 days (6–11), respectively. The procedure’s success was achieved in 100% of patients. The 30-day mortality was 0%. Conclusions: Considering all the limitations related to the small sample, the presented results of a hybrid approach for minimally invasive coronary artery bypass grafting (CABG) appears to be encouraging and acceptable. The main advantage of this approach is related to the reduction of postoperative pain and pulmonary complications.

## 1. Introduction

Coronary artery bypass grafting (CABG) is one of the most effective strategies to improve clinical outcomes in severe coronary artery disease patients [1,2,3,4,5,6]. 

Most of the benefits of CABG surgery are due to the efficacy and long-term patency of the left internal mammary artery (LIMA) to the left anterior descending (LAD) coronary artery. However, CABG performed via sternotomy remains a highly invasive procedure and, despite its excellent long-term results, it is scarcely preferred by patients over transcatheter procedures [7].

In this context, during the last few decades, growing interest has been directed to minimally invasive strategies including robotically assisted CABG, with the final goal of reducing invasiveness, post-operative pain, complications, and hospital stays while maintaining the clinical benefit of CABG procedures [8,9,10,11]. 

Up to now the totally endoscopic coronary artery bypass graft (TECAB) is the most advanced step of a minimally invasive approach. However, it is technically more demanding, and it requires a progressive learning curve. Robotic-assisted minimally invasive direct coronary artery bypass (RA-MIDCAB) surgery has been proposed as an intermediate step between classic open chest CABG and TECAB to minimize the invasiveness and maintain the benefits of a direct hand-sewn anastomosis [12,13,14]. This technique includes the LIMA harvesting with the use of the DaVinci system (DaVinci X surgical system, Intuitive Surgical Inc., Sunnyvale, CA, USA) and the minimally invasive direct coronary artery bypass grafting (MIDCAB) anastomosis through the left anterior mini thoracotomy.

Starting from a robust experience in CABG surgery, a new robotic surgery program was started at our Institution in January 2021 [15].

In this study, we aim to present the results of our initial single-center experience with on-pump arrested heart RA-MIDCAB for surgical myocardial revascularization.

## 2. Materials and Methods

Between January 2021 and October 2022, a total of 17 patients underwent on-pump RA-MIDCAB procedures using the DaVinci system at the University Hospital of Bordeaux (France). The primary indication for surgery included severe left anterior descending artery (LAD) stenosis. Only one patient presented two-vessel disease including LAD and first marginal branch. 

All patients underwent preoperative multi-slice computer tomography (CT) and coronary angiography to assess the LIMA and LAD anatomy, the percentage of coronary stenosis, and to determine which intercostal space to use for the approach. All procedures were performed by one surgeon (L.L.). 

Pre- and post-operative echocardiographic evaluations were performed according to current recommendations [16] by board-certified cardiologists, while the analysis of the surgical outcomes was performed by reviewing electronic health records. 

Post-operative outcomes were defined as occurring within the 30th postoperative day. 

### Operative Set-Up and Strategy

After double-lumen intubation, right internal jugular venous line placement, and a 3D transesophageal echocardiography (TOE) probe were positioned. The left lung was deflated, and the camera trocar was introduced in the fourth or fifth intercostal space (ICS). After insufflation of 10-mmHg carbon dioxide, the camera was inserted, and two additional ports were placed in two ICSs above and below the camera.

**Figure 1:**
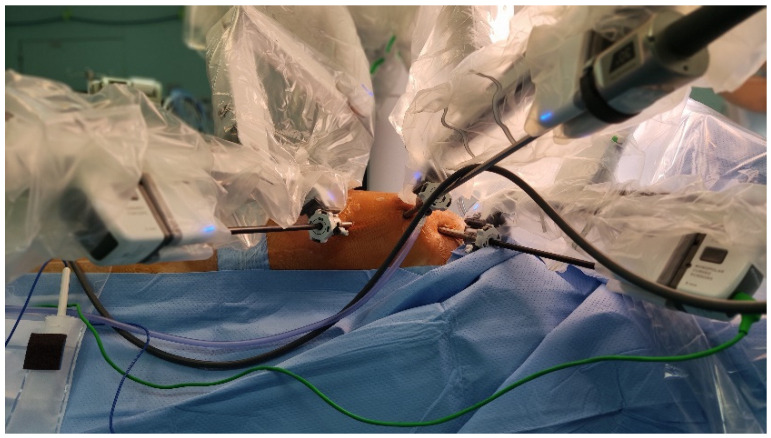
This picture shows the operative setting and the insertion sites of the trocars on the left side of the thorax.

The DaVinci system was then placed on the right side of the patient and robotic arms were inserted into the thorax on the left side of the patient (Figure 1). 

The first step included the pericardial opening, away from the phrenic nerve. This important step allowed the identification of the LAD, the stenosis, and the exact level as to where to perform the anastomosis. Thus, starting from the level of the coronary stenosis, the camera was directed towards the anterior chest wall and a needle inserted on the skin to identify the incision site right in front of the LAD stenosis. The LIMA was harvested in a skeletonized fashion in all patients using robotic monopolar cautery spatula and clips for small branches. 

After systemic heparinization, cardio-pulmonary bypass (CPB) was started according to the standard arterial and venous femoral cannulation technique, and the LIMA was disconnected at its distal end. At this point the DaVinci mechanical arms were extracted from the thorax, a 6-cm skin incision was performed, and a tissue retractor was used to provide exposure through the ICS. 

The antegrade cardioplegia needle/vent was placed in the ascending aorta, and a trans-thoracic aortic clamp was passed through the thorax wall in the second or third ICS in the middle axillary line and positioned around the aorta allowing the aortic clamping and cardioplegic arrest with the injection of cold blood cardioplegia.

After LAD identification and incision, LIMA was anastomosed with a 8–0 prolene running suture.

**Figure 2:**
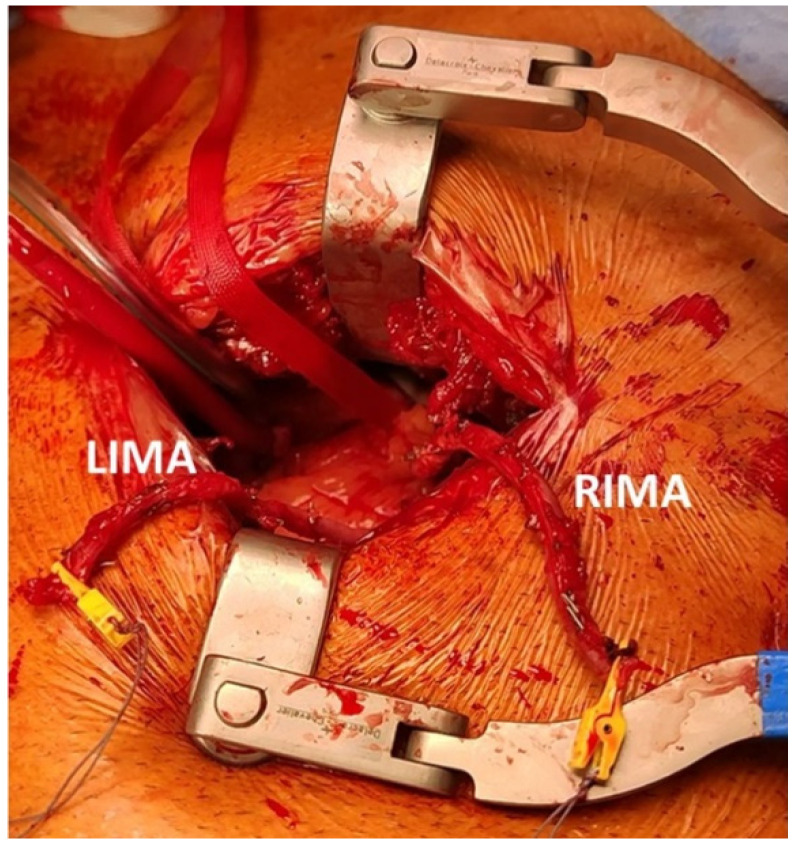
The left mini-thoracotomy approach with two mammary arteries anastomosed in end-to-side fashion. LIMA: left internal mammary artery; RIMA: right internal mammary artery.

Only one patient was treated with two vessel revascularizations with the use of an Y graft combining LIMA with the right internal mammary artery (RIMA). In this case, the robotic arms were placed on the left side of the patient, RIMA and LIMA harvested in a standard fashion and an end-to side anastomosis was performed between the two graft through the direct vision via left mini-thoracotomy (Figure 2).

## 3. Results

A total of 17 patients, 11 males, with a median age of 64 years (56–70), were included in the study. All of these patients were successfully treated with RA-MIDCAB at our center from January 2021 to October 2022. 

Table 1 shows the preoperative characteristics of the patients. Median body mass index (BMI) and EUROscore II were 26.7 kg/m^2^ (24–32) and 0.88% (0.7–1.2), respectively. Most of the patients (65%) were in NYHA functional class II or greater at the time of operation, and median left ventricle ejection fraction was 58% (55–60). None of the patients had previously undergone a cardiac surgery operation.

None of the concomitant procedures were performed. One patient underwent two vessel anastomosis on the LAD and first diagonal branch, and one patient underwent the LAD and first marginal branch revascularization with the use of LIMA and RIMA. The overall median CPB and cross-clamp time were 83 min (76–115) and 38 min (32–58), respectively.

The postoperative details are presented in Table 2.

The median ICU and hospital stays were 2 days (1–4) and 8 days (6–12), respectively.

**Figure 3:**
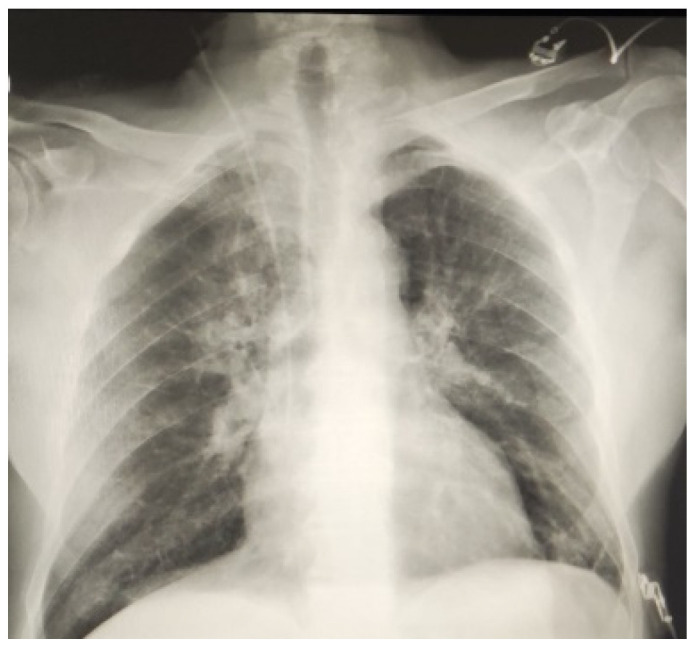
The chest X-ray on a patient during third postoperative day after single vessel RA-MIDCAB. The result appears satisfactory allowing for a faster recovery.

None of the patients reported postoperative bleeding requiring a re-operation, but one patient reported pericardial effusion that was medically treated. One patient who underwent two vessel anastomosis on the LAD and first diagonal branch experienced a postoperative MI during the immediate postoperative phase. Coronary angiography was performed showing occlusion of the first diagonal arterial branch at the level of the anastomosis. The patient well tolerated this event without signs of hemodynamic instability and inotropic support was not needed. For this reason, no invasive procedure was performed, and the patient was discharged to home in fourth postoperative day. We did not report any case of pulmonary effusion or dystelectasis during our experience (Figure 3).

In-hospital and 30-day cardiac-related mortality was 0%.

During the same period considered for the series described, a total of 352 patients underwent standard surgical revascularization via median sternotomy at our center. Although no significant differences were reported in preoperative characteristics between patients who underwent standard CABG and RA-MIDCAB, the median ICU and hospital stay was clearly shorter in the RA-MIDCAB group (4 days (3–5) vs. 2 days (1–4); 11 days (9–13) vs. 8 days (6–12)), respectively. All these results are undoubtedly achieved thanks to the significant reduction in post-operative pain and pulmonary complications related to the use of RA-MIDCAB.

## 4. Discussion

In recent years, minimally invasive surgery has shown a clear superiority compared to classic surgery in reducing functional recovery times, hospitalization length of stay, and complications while maintaining stable results. In addition, it can offer good aesthetic results which is, from a patient’s perspective, an attractive element to accept surgery. For all these reasons, the last two decades were significantly marked by a growing interest in minimally invasive cardiac procedures.

Regarding the treatment of coronary artery disease, there are two main techniques currently in use, MIDCAB and robotic TECAB.

MIDCAB allows for surgical revascularization by means of a left anterior mini-thoracotomy through which both the harvesting of the LIMA and the anastomosis itself are carried out [17]. This is a widely used technique, which significantly reduces the invasiveness of the median sternotomy, allowing both on-pump (with or without a beating heart) and off-pump procedures. The robotic TECAB is considered the last and most technologically advanced step of surgical revascularization, allowing the graft harvesting and the execution of the anastomoses using the robot without making incisions in the chest wall. However, many surgical skills are required to perform both MIDCAB and TECAB, including a relatively long learning curve, a dedicated team, and a high-volume center.

In this paper we wanted to share our initial experience with RA-MIDCAB as an intermediate step combining different techniques for coronary artery revascularization.

Recently, Balkhy et al. [18] demonstrated that beating-heart TECAB is safe and effective with excellent outcomes comparable to standard coronary artery bypass grafting surgery. The authors highlighted that these results can be achieved only with a dedicated experienced team, and when performed frequently.

It appears somewhat clear that starting a new robotic surgery program can be a leap of faith, even for an experienced surgeon who should restart his technical training, facing new instruments, and a new set-up of the procedure. For this reason, before performing TECAB, the learning curve inevitably includes intermediate steps that aim to increase the familiarity with the new devices and procedures. This is exactly what happens with RA-MIDCAB. 

Previous studies demonstrated that TECAB and RA-MIDCAB can be safely performed with excellent short-term clinical outcomes [12,14].

However, TECAB with robotic distal anastomosis procedures could be more expensive than RA-MIDCABs [19].

The first consideration concerning RA-MIDCAB is purely technical: although the LIMA can be taken down manually through a mini-thoracotomy [20], the use of robotics provides total harvesting and also enables dissection of the bilateral IMAs if necessary. In addition, it does not require traumatic maneuvers avoiding the use of retractors that can cause a distortion or fractures of the ribs [21].

The other technical aspect is the possibility of performing an anastomosis through direct vision, having the ability to better control potential bleeding, as well as help from the first assistant if necessary.

The second consideration refers to the need for surgeons to keep the standard of their results high, wanting to ensure the effectiveness of a LIMA to the LAD while minimizing invasiveness. The combination of these techniques is an intermediate step before moving on to the complete execution of the anastomoses through the use of robotics. It allows surgeons to increase the feeling with the device, to accumulate experience, and to know how to manage simple complications. 

In conclusion, minimally invasive coronary surgery is rapidly evolving redesigning the future medical practice of myocardial arterial revascularization. High volume centers should develop specific training programs for future generations of cardiac surgeons considering all available technologies.

Our initial experience confirms that a robotic cardiac surgery program is a step-by-step process with a lot of commitment, attention to details, and continuous training. Careful preoperative evaluation and a dedicated team are the key points for a new robotic surgery program that can also be launched in centers that do not have much experience in minimally invasive surgery.

The main limitation of the study is its retrospective rather than randomized nature. In addition, we are aware that only 17 patients cannot give a solid scientific value to this work. For this reason, further studies with larger samples and longer follow up are needed to demonstrate the efficacy of this technique. 

We know that the RA-MIDCAB here proposed is not the cheaper or less resource-consuming strategy. Nevertheless, we started our journey in robotic cardiac surgery in January 2021 with the main objective of performing mitral procedures, and progressively we also started to use this technology for myocardial revascularization. Although conventional MIDCAB represents a feasible alternative to sternotomy, RA-MIDCAB provides high-definition exposure that minimizes the access trauma of the LITA harvest by avoiding larger incisions and rib spreading, dislocation, or fractures. In addition, in accordance with previous experiences [22], as compared with conventional MIDCAB, the length of the graft is usually longer with the robotic harvesting technology and can be used for bilateral mammary artery revascularization. 

For these reasons, the proposed technique seems to be effective in reducing post-operative pain and pulmonary complications allowing for a reduction in ICU and hospital length of stay.

## Figures and Tables

**Table 1:** Preoperative characteristics.

Variables	Value
Age (years)	64 (56–70)
Gender (male)	11 (65%)
Blood hypertension	15 (88%)
NYHA functional class	
II	9 (53%)
III	1 (6%)
IV	1 (6%)
BMI (kg/m^2^)	27 (24–32)
Dyslipidemia	14 (82%)
COPD	1 (6%)
Smoke	4 (24%)
Atrial fibrillation	1 (6%)
LVEF (%)	58 (55–60)
Previous cardiac surgery	0

Continuous data are reported as median (I, III quartiles); categorical data are reported as a percentage and absolute frequencies. BMI: body mass index; COPD: chronic obstructive pulmonary disease; LVEF: left ventricle ejection fraction; NYHA: New York Heart Association functional class.

**Table 2:** Postoperative characteristics.

Variables	Value
ICU stay (days)	2 (1–4)
Hospital stays (days)	8 (6–12)
Reoperation for bleeding	0
Conversion to sternotomy	0
Pulmonary infection	0
Stroke	0
Acute kidney injury requiring dialysis	0
New onset of atrial fibrillation	1 (6%)
Infective endocarditis	0
30-day overall mortality	0
30-day cardiac-related mortality	0
Postoperative LVEF (%)	60 (55–60)
Postoperative MI	1 (6%)

Continuous data are reported as median (I, III quartiles); categorical data are reported as a percentage and absolute frequencies. ICU: intensive care unit; LVEF: left ventricle ejection fraction; MI: myocardial infarction.

## Data Availability

The data will be shared on reasonable request to the corresponding author.
